# WorldSeasons: a seasonal classification system interpolating biome classifications within the year for better temporal aggregation in climate science

**DOI:** 10.1038/s41597-024-03732-z

**Published:** 2024-08-27

**Authors:** Chris Littleboy, Jens-Arne Subke, Nils Bunnefeld, Isabel L. Jones

**Affiliations:** Biological & Environmental Sciences, Faculty of Natural Sciences, University of Stirling, Stirling, United Kingdom

**Keywords:** Climate sciences, Geography

## Abstract

We present a seasonal classification system to improve the temporal framing of comparative scientific analysis. Research often uses yearly aggregates to understand inherently seasonal phenomena like harvests, monsoons, and droughts. This obscures important trends across time and differences through space by including redundant data. Our classification system allows for a more targeted approach. We split global land into four principal climate zones: desert, arctic and high montane, tropical, and temperate. A cluster analysis with zone-specific variables and weighting splits each month of the year into discrete seasons based on the monthly climate. We expect the data will be able to answer global comparative analysis questions like: are global winters less icy than before? Are wildfires more frequent now in the dry season? How severe are monsoon season flooding events? This is a natural extension of the historical concept of biomes, made possible by recent advances in climate data availability and artificial intelligence.

## Background & Summary

The concept of biomes has been developed and used widely over the past 140 years, including contributions from Köppen (1884), Holdridge (1947), Whittaker (1962) and Olson-Dinerstein (1998) among others^1–5^. The availability of high-resolution monthly climate data in the past 5 years has improved the spatial resolution of biome classifications^6^. Biomes ensure that the spatial frame for comparative analysis is appropriate and data products such as the high-resolution Köppen-Geiger grid^6^, and the ecoregion polygons^7^, are cited by thousands of applied analyses.

A global seasonal classification system is a natural extension of existing biome classification systems. In the same way that there is value in an empirically derived classification system for biomes, there is a value in empirically derived classification systems for seasons. Temperature increases and extreme weather frequency are most commonly reported in yearly composites^8^. Yearly composite satellite data are used to understand large scale Land Use and Land Cover (LULC) change^9–11^. Many phenomena important to the environment and economy are fundamentally seasonal in nature, for example snow melt, monsoons, and harvests^12–14^. For research which compares seasonal phenomena, annual aggregates are an inappropriate temporal frame. Some trends can be captured using annual aggregate data, but these can be diluted by the inclusion of redundant data – information included in the analytical framing which is not relevant to the object of study. Just as biome classifications help define the spatial frame for analysis, season classifications can help define the temporal frame for analysis.

There are formal definitions of seasons. Meteorological seasons divide the year in temperate areas into four equal three-month periods. Astronomical seasons similarly divide temperate areas into four almost equal periods based on solstices and equinoxes. But variation in climate and vegetation, which these classifications purportedly measure, do not come in corresponding, equal intervals; distinct periods of vegetation growth or climate activity that signify a season can be shorter or longer than three months^15^. Furthermore, neither meteorological, nor astronomical seasons accurately describe yearly change in tropics, deserts, or arctic and high montane regions, where there are fewer than four distinct climate windows within an annual cycle^15–17^. Analysts wishing to incorporate intra-annual change are therefore forced to make a difficult choice: to base results on meteorological or astronomical seasons (which do not appropriately capture intra-annual variation and are not appropriate for non-temperate areas), or study change in a small area where seasons can be defined in a locally specific manner^13^.

We present an alternative approach which avoids inappropriate temporal aggregation in studies of climate change. The method outlined harnesses recent advances in the technology and availability of machine learning optimization and Large Language Models (LLMs)^18–20^. Our new seasonal classification groups months into clusters (i.e., seasons) with similar climate and function at high resolution for every location across the globe. Our classification has potential applications for a wide range of analyses.

## Methods

Seasons are generated with a two-tier algorithm which first sorts each location to a climate zone (temperate, tropical, desert, or arctic and high montane) and second sorts each month in that location into a zone-specific season using monthly seasonality data. Underlying data on climate are from three sources. First, WorldClim Version 2, which publishes global raster data on monthly climate^21^. This represents long-term (1970–2000) historical average values for each month. Specifically, we use a temperature variable (the mid-point between monthly minimums and monthly maximum temperature), precipitation, and solar radiation. Second, the European Space Agency’s Climate Change Initiative published a seasonality product of weekly data on vegetation greenness (assessed by the Normalized Difference Vegetation Index, NDVI)^22^. This represents long term (1998–2012) historical values for NDVI each week. This data was temporally aggregated to monthly averages, and spatially resampled to the same grid as the WorldClim raster using the terra package in R^23^. And third, we use the Global Aridity Index^24^. The Aridity Index is a ratio of precipitation to Potential EvapoTranspiration (PET), where PET measures the evapotranspiration of a reference crop (well-watered grass with a height of 12 cm, fixed surface resistance of 70 seconds per meter, and an albedo of 0.23).

We also compute lag variables to determine the size and direction of monthly changes in NDVI and temperature. For example, the January temperature lag variable would be the temperature in January minus the temperature in December. The sign of these lag variables helps determine the difference between, for example, Spring and Autumn^25,26^.

We balanced the need to retain information about complex climate changes, while making the approach as simple as possible – with the fewest possible climate zones and simplest naming conventions for seasons^27^. The process has several core principles:

- Seasons are discrete groups of the year based on complex continuous local changes in weather and the resulting changes in vegetation.
- Temperate areas have 4-season systems (“Winter”, “Spring”, “Summer” and “Autumn”) and lack the extremes present in other climate zones.
- The tropics typically have 2-season systems (“Dry” and “Wet”) and have high rainfall, low intra-year temperature variation, and a minimum yearly average temperature.
- Arctic and high montane regions have two seasons (“Winter” and “Summer”) and have year-long low temperatures.
- Deserts have two seasons (“Cooler” and “Hotter”) and are defined by high aridity (the ratio of rainfall and Potential Evapotranspiration) and low rainfall.
- Apart from in the tropics, seasons must be “temporally contiguous”: once a season ends it cannot reappear in the same annual cycle.
- In the tropics there can be multiple “Wet” seasons due to the Inter-Tropical Convergence Zone (ITCZ)^28^.

### Determining the climate zone

**Fig. 1:**
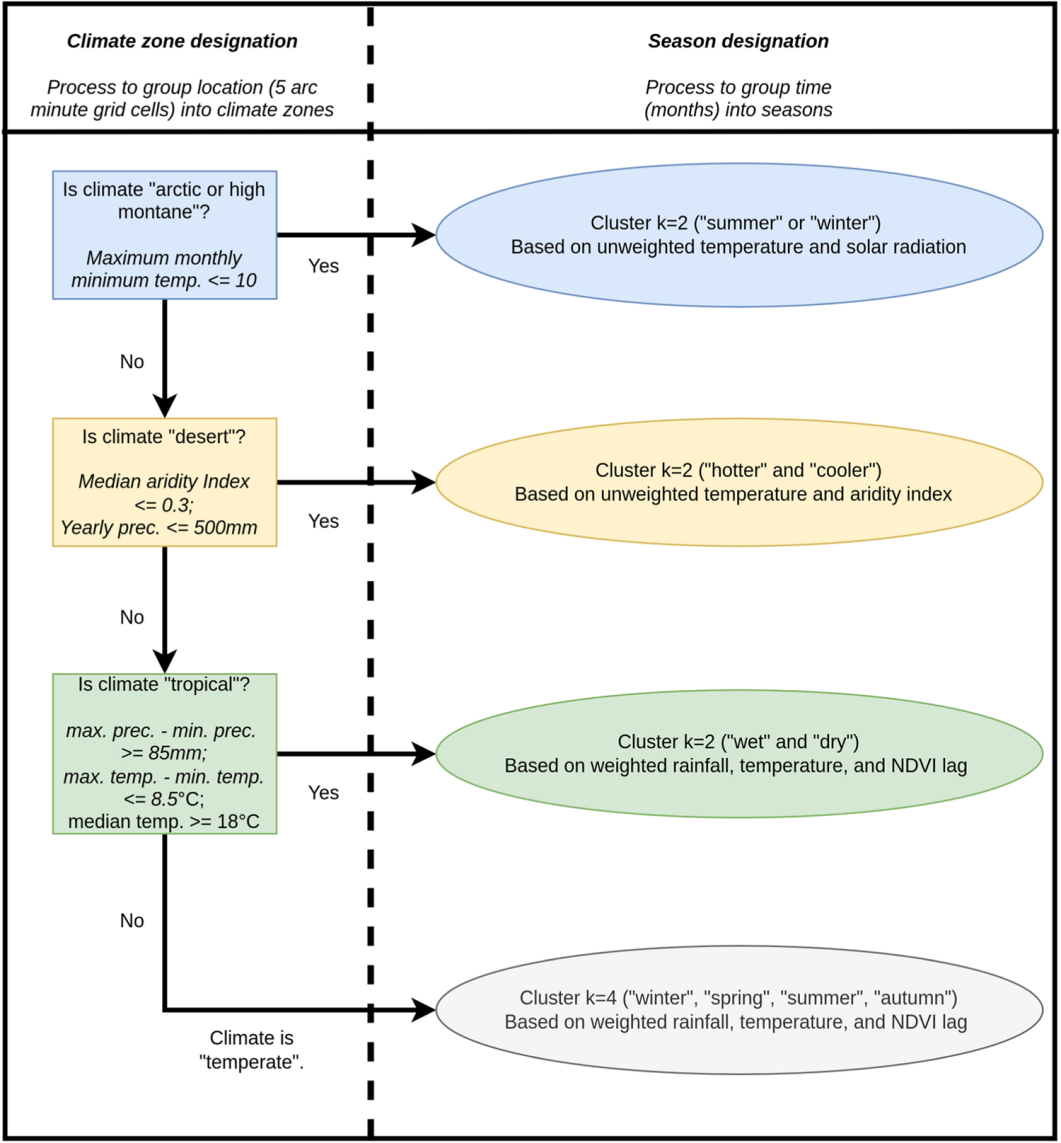
Schematic of the two-stage process to determine climate zones and seasons.

Every location is grouped into one out of four climate zones: arctic and high montane, desert, tropical, and temperate (Fig. 1). The type of climate is determined by several fixed conditions. Arctic climates do not fit into the 4-season systems of temperate climates as Spring and Autumn are too short. Instead, we consider a 2-season system of Winter and Summer as more exact, as in recent studies of polar climates^29^. These climates are defined as areas where the mean minimum temperature of each month in the year never exceeds 10 degrees Celsius, following the approach in the Koppen-Geiger system^6^. This means that a frost can occur on any day of the year, and the growing season is very short. Arctic climates are grouped together with high-montane climates because of the similarities in ecosystem function of these areas^30^.

Deserts are arid and include cold as well as hot deserts^24^. While deserts can be distinguished from other climate zones using aridity, seasons within desert climates are mainly defined by changes in temperature^31^. This is particularly the case for the arctic dry tundra, Tibetan plateau, and the Gobi, which have arid environments with extreme intra-annual temperature changes^32^. Most deserts, such as the Sahara, have a more consistent extreme heat.

Tropical regions are determined by three thresholds: first, the intra-annual difference in rainfall (difference between the rainiest month and driest month). Second, the intra-annual difference in temperature (difference between the hottest and coldest month). These two thresholds are parameters set during optimization. Third, a threshold of temperature was used to exclude areas which are cold year-round, but have little seasonal temperature variation, which is possible due to ocean currents^33^. Where the difference between the wettest and driest months is high, the difference between the hottest and coldest month is low, and the average temperature is consistently high, areas are considered to be tropical.

The remaining areas are not characterized by such extremes and are designated as temperate.

### Cluster analysis

We use a k-means cluster analysis to determine monthly seasons for each location: a 5-arc minute grid cell. The process is the same for locations in each climate zone, but the resulting seasons depend on the underlying climate data. For arctic and high montane climates, we use an un-weighted 2D cluster analysis (temperature, solar radiation) with two centers (Summer, Winter). For desert climates, we use an un-weighted 2D cluster analysis (temperature, aridity) with two centers (Hotter, Cooler). Our variables selection and weighting strategy was aimed to capture key seasonal trends in different climate zones with the minimum possible input data. In un-weighted cluster analysis each variable has equal importance. In deserts, and in arctic and and high montane climates, unweighted cluster analysis is appropriate because input variables are positively correlated and correspond with stark seasonal differences. One exception is equatorial deserts where the temperature differences between “Cooler” and “Hotter” are minimal, and seasonal analysis of change is less important. Weighting is necessary for the tropical and temperate climate zones. For temperate climates, we use a weighted 4D cluster analysis (rainfall, temperature, NDVI lag and temperature lag) with four centers (Winter, Spring, Summer, Autumn). For tropical climates, we use a weighted 3D cluster analysis (rainfall, temperature, NDVI lag) with two centers (Wet, Dry). The temperature lag was excluded from tropical areas because the direction of month-to-month temperature change is often not a continuous function in the tropics.

We weight the variables because some climate variables are dominant in distinguishing seasons in specific locations. Weighting ensures that these key variables were more (or less) important in determining the seasonal designation for each month. The strategy to weight the variables involved two processes. First, we generated reference data of month-season pairs for global cities. Data with a local understanding of how the seasons change through the year, on a global scale, did not already exist. We generated this using the Large Language Model ChatGPT 3.5^34,35^. Second, we optimized our algorithm to produce seasonal designations from the raw climate data which matched seasonal designations in the reference data as closely as possible.

### Reference data

We gathered point locations for cities throughout the world using naturalearth data^36^. To ensure appropriate geographic representation, we selected 10 cities at random in each country. For each city-country pair we ran the prompt in ChatGPT:

“What is the season for each month in [city-country]. Do not include any introduction. Respond in the format: January,xxx, February,xxx, March,xxx, April,xxx, May,xxx, June,xxx, July,xxx, August,xxx, September,xxx, October,xxx, November,xxx, December,xxx; Do not include line breaks.”

The raw output of this shows the heterogeneity in local definitions of seasons. Especially in the tropics, there were many different names for “Dry” season and “Wet” season.

**Fig. 2:**
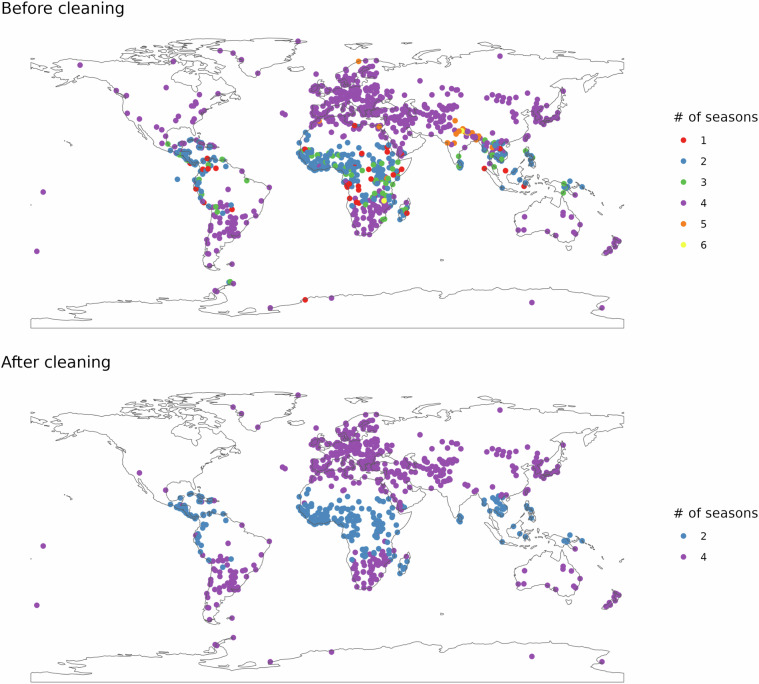
Seasonal simplification by recategorizing tropical seasons to Wet and Dry. The upper map shows the number of seasons in each city before cleaning the ChatGPT data. The lower map shows the number of seasons in the cleaned data.

Seasons in Tropical areas relate to volume of rainfall, and we forced a simpler 2-season category on tropical areas (Fig. 2). Often, due to the ICTZ, there are two separate “Wet” seasons during the year which can have different names. As our approach aimed for simplification, we adjusted labels to either “dry” or “wet” (Table 1).

**Table 1:** Recoding of seasons from raw ChatGPT data to the clean and simple version.

Gpt output	Cleaned label	Frequency
Dry Season	dry	468
Hot and Dry	dry	30
Harmattan	dry	22
Warm and Dry	dry	10
Hot	dry	10
Sunny and Dry	dry	7
Cool and Dry	dry	4
Rainy	wet	306
Wet Season	wet	259
Rainy Season	wet	193
Wet-Season	wet	20
Warm and Humid	wet	10
Hot and Wet	wet	10
Hot and Humid	wet	10
Short Rainy Season	wet	8
Rain	wet	6
Humid	wet	5
Warm and Wet	wet	5
Long Rainy Season	wet	4

K-means was chosen over other clustering algorithms because of its speed and because it is a well-established algorithm. Since we have pre-determined the number of discrete seasons in each climate zone, the typical downside of K-means clustering – that analysts need to pre-define the number of clusters in the data – becomes a strength^37^. In order that each variable has equal importance irrespective of the reported unit (mm of rain, degrees Celsius), variables are normalized by subtracting the mean and dividing by the standard deviation. In two of the climate zones – arctic and high montane and deserts – variables are not weighted. This is because we only include two variables in the cluster analysis for these climate zones – in the desert climate zone, variables are temperature and aridity, and in arctic and high montane climate zone, variables are temperature and solar radiation. In both cases these are correlated and weighting the variables is not important for cluster selection.

In tropical and temperate regions weights are more important to achieve seasons which align with current understanding. While tropical seasons are defined more by seasonal differences in rainfall, temperate seasons are defined more by temperature changes and the resulting changes in growing conditions. As such, certain climate variables are more important than others in such conditions. These variables are weighted higher, and the weights were determined using *irace* – an R package for automatic algorithm tuning. The parameter space – 9 variables of bounded real numbers – is sufficiently large to not allow for an exhaustive search approach to determining weights^38^. All parameters in the optimization algorithm are bounded real numbers. The intra-annual temperature and precipitation difference thresholds for determining whether a location is tropical or temperate were bound between 3 and 14 degrees Celsius, and 80 mm and 130 mm of precipitation. Introducing these bounds was done to help minimize computation time following several initial runs of the optimization algorithm which returned values within this range. Both sets of bounds were deliberately flexible – large enough to allow for smaller and larger than expected values from the optimization search, but small enough to help the optimization achieve convergence. In addition, all weights were bound between 0.25 and 4 so that a variable could range from a quarter as important to four times as important as an unweighted variable in determining the cluster. These bounds were set to provide balance and prevent any one variable becoming too dominant in the determination of seasons. which can lead to chosen weights which work well in many contexts and poorly in others.

We defined a simple error function where 1 was added to the error for each month that the cluster algorithm produced a season different from the ChatGPT season. If the algorithm decided that a temperate area (as determined by ChatGPT) was tropical, the error was capped at 6. This was set to be a high penalty for categorizing a tropical city in the reference data as a temperate city in the algorithm. All settings were calibrated by visual assessment of the seasonal classification performance and the authors’ knowledge of certain geographies. The optimization process aims to avoid such subjectivity in the actual choice of weights.

The optimization trained on 70% of the cities in the ChatGPT reference data – totaling 573 locations. We then tested the performance of five “elite” configurations on the remainder of the reference data – totaling 245 cities. Final parameters were selected from the training set with the lowest mean error on test data (Tables 2, 3, and 4).

**Table 2:** Thresholds to determine whether a grid cell is considered temperate or tropical.

Variable	Value
Precipitation (mm)	81.972
Temperature (°C)	7.52

Differences are maximum monthly values minus minimum monthly values for each climate variable. Values above the precipitation difference threshold and below the temperature difference threshold are tropical.

**Table 3:** Optimized weights for temperate areas.

Variable	Value
Temperature	3.521
Precipitation lag	3.237
Temperature lag	2.031
NDVI lag	0.312

In the k-means cluster analysis, climate variables in temperate areas are weighted with these values to determine the season in each month.

**Table 4:** Optimized weights for tropical areas.

Variable	Value
Temperature	3.958
Precipitation lag	3.72
NDVI lag	1.713

In the k-means cluster analysis climate variables in tropical areas are weighted with these values to determine the season in each month. The temperature lag variable is excluded because the direction of month-on-month temperature changes is not a continuous function like in temperate areas.

The mean error of the final optimized parameters was 2.3, meaning that for each location there were 2.3 months of the year with different seasonal designations between our algorithm and the reference data.

Table 5 shows the tabulated errors which illustrate a skew: our algorithm typically either matched the ChatGPT data completely or did not match at all. All cities where none of the months matched were in the tropics and were typically coastal or had a higher elevation. This is due mainly to seasons which were inaccurately assigned to these unique climates in the ChatGPT reference data. Of the cities where the months partially matched, this was typically due to an offset effect. For example, our algorithm often considered all seasons of the year to begin one month later.

**Table 5:** Frequency of error scores for the final chosen parameter configuration.

Error	Frequency
0	101
1	14
2	38
3	42
4	21
5	6
6	3
7	4
8	1
9	1
10	2
12	12

Overall, we consider this mean error to be sufficiently low to trust in the weighting process. No fixed set of parameters can eliminate errors between the ChatGPT reference data and predicted seasonal classifications. By having a fixed set of parameters we ensure global consistency in the approach to determine seasons. This is a core purpose for this data. And by choosing fixed parameters that minimize classification errors with respect to the ChatGPT dataset, we ensure that our seasonal classifications match local understandings of seasons as closely as possible.

### Post processing

Seasons are collected for each grid cell using a cluster analysis with the weights determined by the optimization process. Where a month does not clearly belong to a specific season, perhaps towards the end of winter and the beginning of spring, the unprocessed raster can show alternating seasonal designations within a small region. The *focal* function in the *terra* R package helps to smooth the transitions between landscapes. Finally, we reproject the data to the pseudo cylindrical Equal Earth projection^39^. This ensures that areas for each grid cell are equivalent globally.

## Data Records

**Fig. 3:**
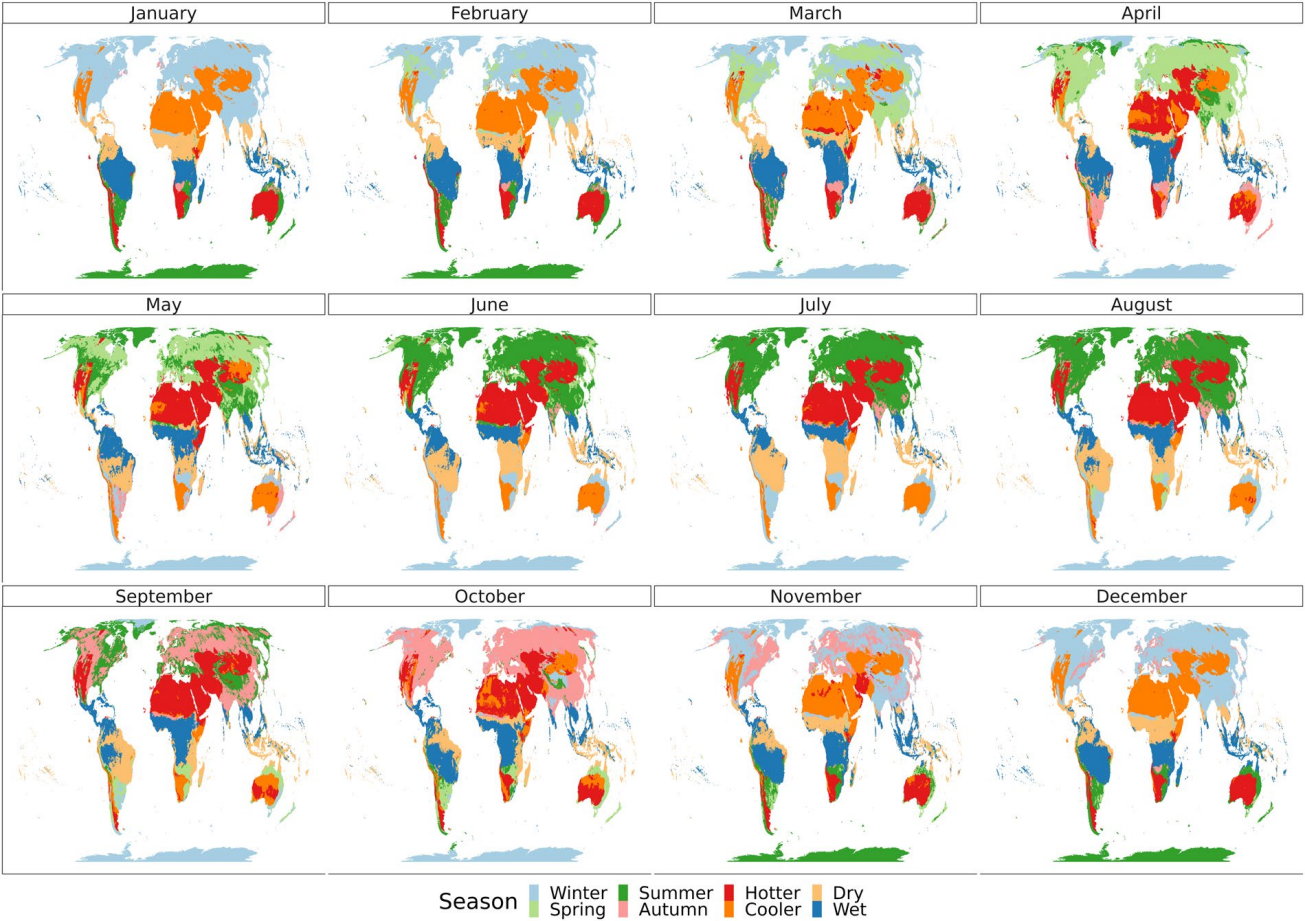
The twelve panels show the results of the seasonal classification for each month.

**Fig. 4:**
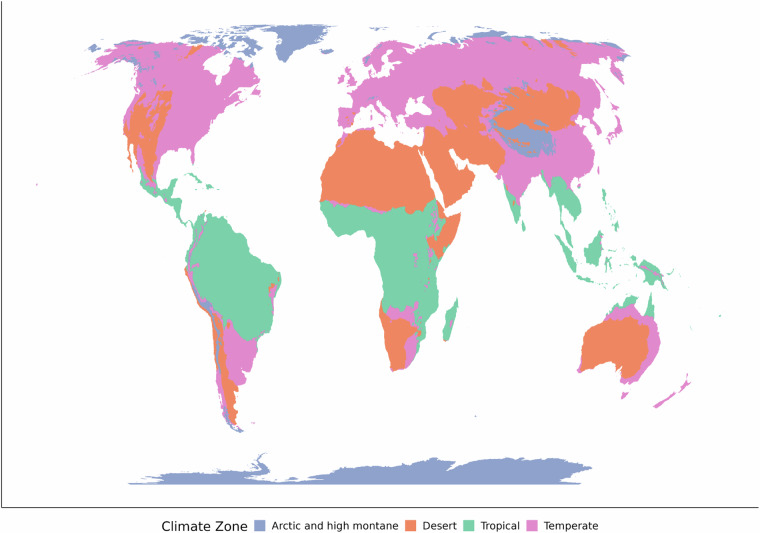
Output of applying thresholds to each grid cell of the underlying climate data to determine the four Climate Zones.

Our data is publicly available for download^40^. For every 8 km x 8 km cell on land we have a 2 × 12 matrix showing month–season pairs, as shown in Fig. 3. The file is available to download in .tif format, with twelve layers. It is also available as a .txt file, with four columns: x, y (representing longitude and latitude in the Equal Area projection), month, and season. We also include our Climate Zone data, available as a single layer .tif file, as shown in Fig. 4.

All code and data used to produce the final file is either freely available within this submission or available for download from the original publishers. We are releasing the data with a CC-BY license.

## Technical Validation

Validation of the model was an integral part of the development of the data through the optimization of weights and thresholds using the ChatGPT reference data. Due to the subjective nature of the concept being quantified, it is difficult to validate beyond this^41^. As with biome classifications, the best approach will reflect the context in which the classification is used^42^. We anticipate that future versions of the model may be necessary to adapt to different use-cases.

## Data Availability

All code used to produce and visualize the data is available. The code is fully documented. It relies on several R packages^19,23,35,43–45^. Our ChatGPT reference data cannot be reproduced due to the nature of large language models. Nevertheless, we include the code to produce and clean the data, as well as our final reference data in .csv format. Likewise, the analysis to find optimal weights can produce different results at each running. We include the code to run this optimization, though there are some system-specific setup requirements. The processing required a machine with large RAM capacity. Please feel free to contact the corresponding author if there are any questions regarding the code.
